# CDK4/6 Inhibition Reprograms Mitochondrial Metabolism in BRAF^V600^ Melanoma via a p53 Dependent Pathway

**DOI:** 10.3390/cancers13030524

**Published:** 2021-01-29

**Authors:** Nancy T. Santiappillai, Shatha Abuhammad, Alison Slater, Laura Kirby, Grant A. McArthur, Karen E. Sheppard, Lorey K. Smith

**Affiliations:** 1 Cancer Research Division, Peter MacCallum Cancer Centre, Melbourne 3052, Australia; nancy.santiappillai@sydney.edu.au (N.T.S.); shatha@gmx.com (S.A.); alison.slater@petermac.org (A.S.); laura.kirby@petermac.org (L.K.); grant.mcarthur@petermac.org (G.A.M.); 2 Department of Biochemistry and Molecular Biology, University of Melbourne, Melbourne 3052, Australia; 3 Sir Peter MacCallum Department of Oncology, University of Melbourne, Melbourne 3052, Australia

**Keywords:** melanoma, targeted therapy, BRAF, CDK4, metabolism

## Abstract

**Simple Summary:**

Cyclin-dependent kinases 4 and 6 (CDK4/6) are key enzymes controlling the cell cycle. CDK4/6 inhibitors are being tested in multiple clinical trials for a range of cancers including melanoma, and a deeper understanding of how they interact with other therapies is vital for their clinical development. Beyond the cell cycle, CDK4/6 regulates cell metabolism, which is a critical factor determining response to standard-of-care mitogen-activated protein kinase (MAPK) pathway therapies in melanoma. Here, we show that CDK4/6 inhibitors increase glutamine and fatty acid-oxidation-dependent mitochondrial metabolism in melanoma cells, but they do not alter the metabolic response to MAPK inhibitors. These observations shed light on how CDK4/6 inhibitors impinge on the regulation of metabolism and how they interact with other therapies in the setting of melanoma.

**Abstract:**

Cyclin-dependent kinase 4 and 6 (CDK4/6) inhibitors are being tested in numerous clinical trials and are currently employed successfully in the clinic for the treatment of breast cancers. Understanding their mechanism of action and interaction with other therapies is vital in their clinical development. CDK4/6 regulate the cell cycle via phosphorylation and inhibition of the tumour suppressor RB, and in addition can phosphorylate many cellular proteins and modulate numerous cellular functions including cell metabolism. Metabolic reprogramming is observed in melanoma following standard-of-care BRAF/MEK inhibition and is involved in both therapeutic response and resistance. In preclinical models, CDK4/6 inhibitors overcome BRAF/MEK inhibitor resistance, leading to sustained tumour regression; however, the metabolic response to this combination has not been explored. Here, we investigate how CDK4/6 inhibition reprograms metabolism and if this alters metabolic reprogramming observed upon BRAF/MEK inhibition. Although CDK4/6 inhibition has no substantial effect on the metabolic phenotype following BRAF/MEK targeted therapy in melanoma, CDK4/6 inhibition alone significantly enhances mitochondrial metabolism. The increase in mitochondrial metabolism in melanoma cells following CDK4/6 inhibition is fuelled in part by both glutamine metabolism and fatty acid oxidation pathways and is partially dependent on p53. Collectively, our findings identify new p53-dependent metabolic vulnerabilities that may be targeted to improve response to CDK4/6 inhibitors.

## 1. Introduction

Mitogen-activated protein kinase (MAPK) cascades are crucial for cell signalling and have been found to control the growth and survival of a wide range of human tumours [1]. BRAF, a component of the MAPK signalling pathway, is mutated in approximately 40% of melanomas, and of these, approximately 90% are at codon 600 and involve the substitution of valine to glutamic acid [2,3]. BRAF and MEK inhibitors have been developed as a breakthrough treatment for BRAF mutant melanoma patients; however, the development of resistance after a median progression-free survival of just over 12 months in patients with advanced disease remains a clinical challenge [4,5].

In addition to the MAPK pathway, deregulation of cyclin-dependent kinase 4 and 6 (CDK4/6) activity frequently occurs in melanoma, mainly due to inactivation of the CDK4/6 inhibitor p16^INK4A^ via gene deletion, somatic mutation, or promoter hypermethylation [6,7]. Moreover, CDK4/6 are key downstream targets of MAPK signalling [8]; therefore, the CDK4/6 pathway has emerged as a potential therapeutic target for treating melanoma patients. The high frequency of CDK4/6 pathway activation in cancer more broadly has led to the development of CDK4/6 inhibitors, such as palbociclib, ribociclib and abemaciclib [9], and the therapeutic effects of palbociclib have been studied in the context of BRAF inhibitor resistance in melanoma. Importantly, BRAF inhibitor-resistant tumours remain sensitive to palbociclib treatment [7,10], while palbociclib in combination with a BRAF inhibitor induces rapid and sustained tumour regression in vivo without acquired resistance [11]. Mechanisms underpinning this synergy remain unclear.

Cancer cells display alterations in energy metabolism to allow increased cell division and growth [12]. Oncogenic BRAF elicits metabolic reprogramming in melanoma cells consistent with the classical Warburg effect, whereby increased aerobic glycolysis and decreased reliance on oxidative metabolism are observed. These effects have been shown to be mediated by a network of transcription factors including HIF1α and c-MYC, which leads to increased expression of the glucose transporters GLUT1 and GLUT3 and subsequent upregulation of glycolysis [13]. Importantly, these metabolic effects are reversed upon BRAF inhibition, and BRAF inhibitor sensitivity correlates with the degree of inhibition of the glycolytic response [13]. Interestingly, BRAF inhibition also leads to a compensatory increase in mitochondrial biogenesis and oxidative phosphorylation (OXPHOS), driven at least in part by activation of PGC1α [14], a master regulator of mitochondrial biogenesis. This adaptive mitochondrial reprogramming by melanoma cells likely occurs as a survival mechanism to rescue the cells from suppressed glycolysis. Linking these metabolic events to therapeutic response in patients, PGC1α expression is induced in BRAF^V600^ melanoma patients treated with BRAF inhibitors, either alone [14] or in combination with MEK inhibitors [15], whilst an elevated mitochondrial biogenesis signature is observed in tumours that relapse following MAPK inhibitor treatment [16].

In recent years, a link between metabolic pathways and cell-cycle progression has also been observed, and several direct targets of CDK4/6 are involved in metabolism. Lopez-Mejia and colleagues [17], using mouse fibroblasts, found that CDK4 via phosphorylation of AMPKa2 decreases fatty acid oxidation and increases anaerobic glycolysis. Consistent with a role for CDK4/6 signalling in glycolysis, either depletion or inhibition of CDK4/6 enhances glucose, as well as glutamine and amino acid metabolism, in RAS-mutated HCT116 colorectal cancer cells [18] and increases glycolytic and oxidative metabolism in pancreatic ductal adenocarcinoma cells [19]. The role of CDK4/6 in metabolism is context-dependent, and in some cases also appears to occur independently of its role in cell cycle progression. Lee and colleagues found that insulin-dependent activation of CDK4 in mice suppresses hepatic glucose production via phosphorylation of the histone acetyletransferase GCN5 [20]. This in turn inhibits PGC1α activity on gluconeogenic genes independently of cell cycle progression. Collectively, these data highlight a role for CDK4/6 in metabolic reprogramming in a range of contexts and cancer settings; however, there has been no previous report of how CDK4/6 regulates metabolism in the setting of melanoma, nor of how CDK4/6 influences the metabolic response to BRAF/MAPK pathway targeted therapies.

Based on the importance of metabolism in targeted therapy response in melanoma, we characterise how CDK4/6 inhibition reprograms metabolism in BRAF^V600^ melanoma, alone and in combination with BRAF and MEK inhibitors. Using BRAF mutant melanoma cell models, we demonstrate that although CDK4/6 inhibition does not alter the metabolic phenotype following BRAF/MEK inhibition, CDK4/6 inhibition alone significantly enhances mitochondrial metabolism. This increase in mitochondrial metabolism is fuelled by both glutamine metabolism and fatty acid oxidation pathways, and we demonstrate that this is mediated, at least in part, by a p53-dependent pathway. Collectively, our findings identify new p53-dependent metabolic vulnerabilities that may be targeted to improve response to CDK4/6 inhibitors in melanoma.

## 2. Results

### 2.1. CDK4/6 Inhibition Increases Mitochondrial Metabolism but Not the Response to BRAF/MEK Inhibition

**Figure 1:**
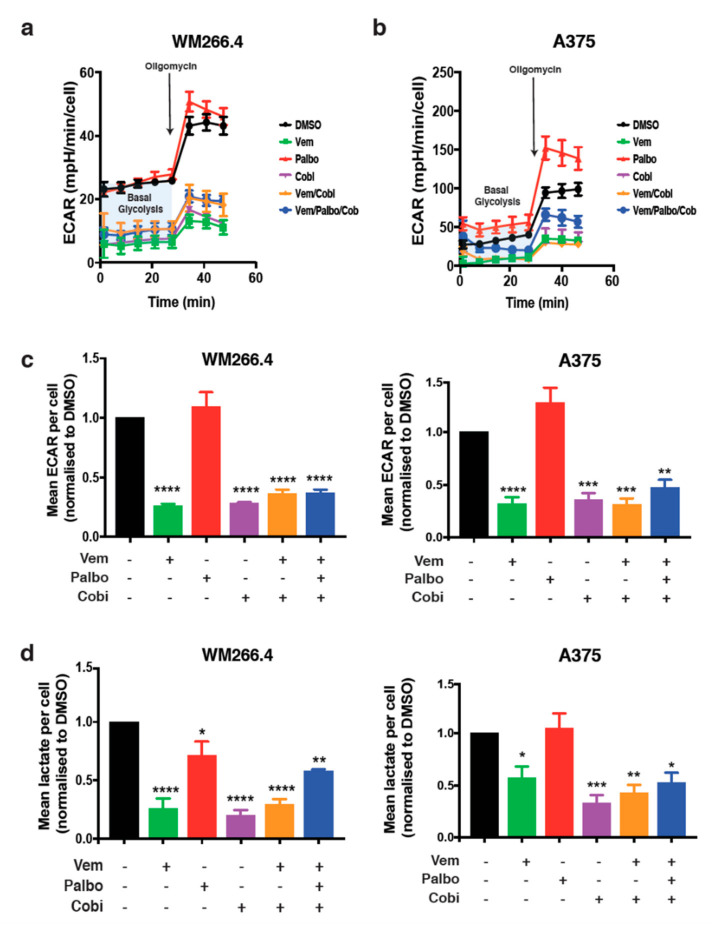
Palbociclib treatment has no substantial effect on the glycolytic phenotype of vemurafenib and cobimetinib. Extracellular acidification rate (ECAR) was determined using Seahorse Extracellular Flux analysis in WM266.4 (**a**) and A375 (**b**) cells, treated for 72 h as indicated with palbociclib (palbo; 1 μM), vemurafenib (Vem; 300 nM), cobimetinib (cobi; 10 nM) or their combination. Representative ECAR profiles are shown (from 3 independent experiments). (**c**) Basal ECAR in WM266.4 and A375 cells treated for 72 h as indicated. (**d**) Lactate production per cell in WM266.4 and A375 cells treated for 72 h as indicated. Data are normalised to cell number and expressed as fold change relative to DMSO controls. Error bars ± SEM, *n* = 3. Statistical significance was determined by one-way ANOVA: * *p* = 0.05–0.01, ** *p* = 0.01–0.001, *** *p* = 0.001–0.0001, **** *p* < 0.0001.

To investigate whether CDK4/6 inhibition alone or in combination with BRAF and MEK inhibition alters metabolic pathways in melanoma, we utilised the BRAF mutant melanoma cell lines WM266.4 and A375 and specific inhibitors of CDK4/6 (palbociclib), BRAF (vemurafenib), and MEK (cobimetinib). Activity of these drugs in WM266.4 and A375 cells was confirmed using Western blot analysis of phospho-ERK and phospho-RB (Figure S1a), and drug sensitivity was determined using dose–response assays (Figure S1b). Calculation of the mean GI50 (the concentration of each drug that results in 50% growth inhibition) revealed high sensitivity of both cell lines to each individual drug (Figure S1c), with slight differences in the sensitivity to vemurafenib and palbociclib observed between the cell lines (A375 cells are less sensitive to palbociclib, whilst the WM266.4 cells are less sensitive to vemurafenib). We have previously shown that palbociclib in combination with a BRAF inhibitor enhances the anti-proliferative effects of each drug [11], and we have extended these observations to include the BRAF/MEK inhibitor combination, alone or in combination with palbociclib. Although robust antiproliferative effects are observed in cells treated with the BRAF/MEK inhibitor combination, this was further exacerbated with the addition of palbociclib (Figure S1d). We also observed a consistent and significant increase in cell death in the BRAF/MEK inhibitor-treated cells; however, this was not further increased by the addition of palbociclib (Figure S1e). We also saw no evidence of decreased cell viability in the cells treated with palbociclib alone, in line with the predominant cytostatic effects of these inhibitors. Having established the sensitivity and proliferative response to vemurafenib, cobimetinib and palbociclib, and their combinations, we next explored the effect of these drugs on metabolism in the A375 and WM266.4 cells. In line with previous studies [14], vemurafenib and cobimetinib or their combination significantly decreased extracellular acidification rates (ECAR) and lactate production, demonstrating that these inhibitors induce a decrease in glycolysis (Figure 1a–d). In contrast, palbociclib alone had no effect on ECAR or lactate production and did not alter the response to the combination of vemurafenib and cobimetinib (Figure 1a–d). We also assessed ECAR measurements post-injection with the mitochondrial inhibitor oligomycin as an indicator of compensatory glycolysis (Figure 1a); however, no consistent effects were observed in palbociclib-treated cells. Consistent with these data, vemurafenib and cobimetinib, but not palbociclib, decreased the expression of glycolysis-associated genes and proteins (Figure S2). Together, these data suggest that CDK4/6 activity does not alter glycolysis in BRAF mutant melanoma cells, nor does it effect the glycolytic response following MAPK pathway inhibition.

**Figure 2:**
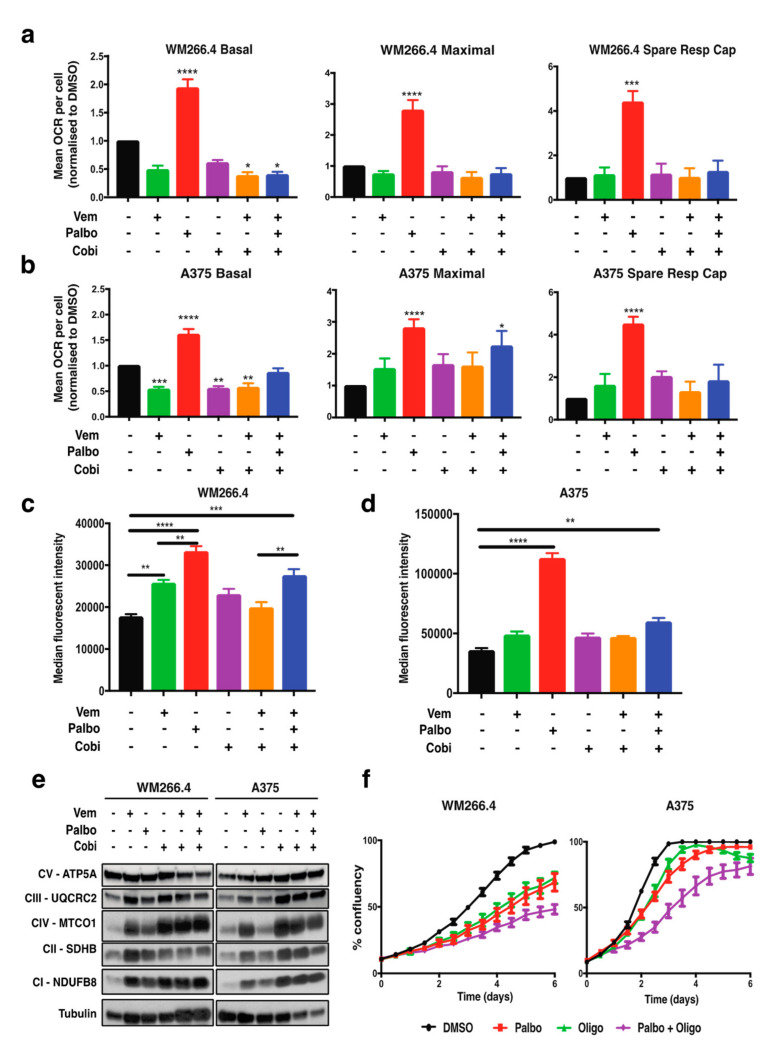
Palbociclib increases mitochondrial respiration in melanoma cells. (**a**,**b**) Oxygen consumption rate (OCR) was determined using Seahorse extracellular flux analysis in WM266.4 and A375 cells treated as indicated for 72 h with palbociclib (palbo; 1 μM), vemurafenib (vem; 300 nM), cobimetinib (cobi; 10 nM) or their combination. Data are normalised to cell number and expressed as fold change relative to DMSO controls. (**c**,**d**) Mitochondrial mass and activity were determined as the median fluorescent intensity of Mitotracker staining measured using FACs analysis in WM266.4 and A375 cells treated as indicated for 72 h. (**e**) WM266.4 and A375 cells were treated as indicated for 72 h, and protein lysates were assessed for the indicated proteins using Western blot analysis. (**f**) WM266.4 and A375 cells were treated with palbo (1 μM) and/or oligomycin (1 μM) for 6 days, and percent confluency was analysed over time using an IncuCyte to assess cell proliferation. Data is representative of 3 biological replicates. Error bars ± SEM, *n* = 3. Statistical significance was determined by One-way ANOVA: * *p* = 0.05–0.01, ** *p* = 0.01–0.001, *** *p* = 0.001–0.0001, **** *p* < 0.0001.

We have previously demonstrated [13] that treatment with vemurafenib for 24 h induces a small but significant decrease in basal oxygen consumption rate (OCR), and our current studies both confirm and extend this observation by demonstrating that cobimetinib and the combination of vemurafenib plus cobimetinib also decrease basal OCR at 72 h post-treatment (Figure 2a,b and Figure S3). In contrast, palbociclib induced a significant increase in basal OCR, maximal OCR, and the spare respiratory capacity; however, this was lost with the addition of vemurafenib and cobimetinib (Figure 2a,b and Figure S3). Consistently, functional mitochondrial mass, as determined by Mitotracker staining, was also increased by palbociclib, but the response was diminished with the addition of vemurafenib and cobimetinib (Figure 2c,d). To investigate the palbociclib-driven changes in mitochondrial metabolism, we examined mRNA expression or protein levels of key regulators of mitochondrial biogenesis in melanoma (MITF, PGC1α and TFAM) [14,16] (Figure S2) as well as protein levels of key components of the five mitochondrial OXPHOS complexes (Figure 2e). As expected, analysis of MITF protein levels and PGC1α gene expression showed an increase in response to vemurafenib and cobimetinib and their combination but no change in MITF protein levels, and no consistent change in PGC1α mRNA levels was observed following palbociclib treatment in the A375 and WM266.4 cells (Figure S2). In contrast, protein levels of four out of five OXPHOS proteins showed an increase in expression with all treatments (Figure 2e); however, this occurred to a lesser extent with palbociclib. Importantly, these results suggest that CDK4/6 inhibition reprograms mitochondrial metabolism and induces mitochondrial biogenesis in melanoma cells, in agreement with studies in other cancer models [18,19]. However, the underlying mechanisms appear to be distinct to those following MAPK pathway inhibition, which are predominantly mediated via the MITF-PGC1A and TFAM pathways [14,16]. Furthermore, palbociclib does not appear to exacerbate the mitochondrial reprogramming observed following BRAF/MEK inhibition; however, this combination diminishes the palbociclib-induced increase in mitochondrial metabolism.

Due to the observed increase in mitochondrial metabolism and mass following palbociclib treatment alone, the role of mitochondrial metabolism in palbociclib sensitivity in melanoma cells was further explored. Mitochondrial metabolism was inhibited with oligomycin, an inhibitor of the mitochondrial enzyme ATP synthase. Cells were treated with oligomycin, alone or in combination with palbociclib. Notably, a decrease in cell proliferation was observed in cells treated with the combination of oligomycin and palbociclib compared to palbociclib alone (Figure 2f), indicating that inhibition of mitochondrial metabolism can increase the sensitivity of BRAF mutant melanoma cells to palbociclib. However, given that the increase in OXPHOS protein levels was not as substantial or consistent as observed following BRAF and MEK inhibition (Figure 2e), these data suggest that increased mitochondrial metabolism following CDK4/6 inhibition is not primarily driven by an increase in OXPHOS complex activity itself, but may result from upregulation of an alternative pathway that fuels mitochondrial metabolism.

### 2.2. CDK4/6 Inhibition Alters Glutamine Metabolism and Fatty Acid Oxidation in BRAF^V600^ Melanoma Cells

**Figure 3:**
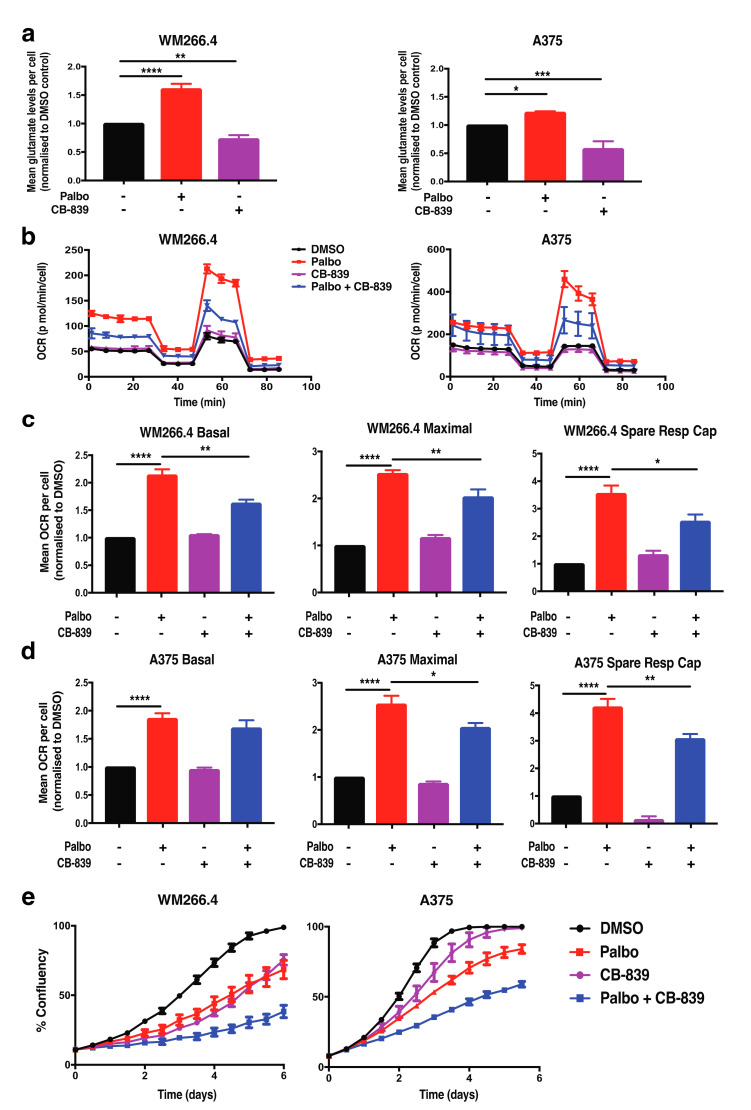
Increased mitochondrial metabolism following palbociclib treatment is partially dependent on glutamine metabolism. (**a**) Intracellular glutamate levels were assessed in WM266.4 and A375 cells treated for 72 h with CB-839 (200 nM) and palbociclib (Palbo; 1 μM) as indicated. Data are normalised to cell number and expressed as fold change relative to DMSO controls. (**b**) Oxygen consumption rate (OCR) was determined using Seahorse extracellular flux analysis in WM266.4 and A375 cells treated for 72 h as indicated. Data show a representative profile from 3 independent experiments. (**c**,**d**) Basal OCR, maximal OCR and spare respiratory capacity were determined from OCR profiles shown in (**b**) for WM266.4 (**c**) and A375 (**d**) cells. Data are normalised to cell number and expressed as fold change relative to DMSO controls. (**e**) WM266.4 and A375 cells were treated with palbo (1 μM) or CB-839 (200 nM) for 6 days, and percentage confluency was analysed over time using an IncuCyte to monitor cell proliferation. Data is representative of 3 biological replicates. Error bars ± SEM, *n* = 3. Statistical significance was determined by One-way ANOVA: * *p* = 0.05–0.01, ** *p* = 0.01–0.001, *** *p* = 0.001–0.0001, **** *p* < 0.0001.

Glutamine feeds into the tricarboxylic acid (TCA) cycle within the mitochondria to fuel oxidative metabolism and has been shown to drive CDK4-dependent metabolic reprogramming within other cancer types [19]. In order to assess a role for glutamine metabolism in metabolic reprogramming following CDK4/6 inhibition in melanoma, we first assessed the levels and activity of a key enzyme involved in glutamine metabolism, glutaminase (GLS1). Although GLS1 protein levels remained unchanged following palbociclib treatment (Figure S4a), intracellular glutamate levels were significantly increased in both cell lines (Figure 3a), indicating increased glutamine pathway activity and a potential role for glutamine metabolism in mitochondrial reprogramming following CDK4/6 inhibition in melanoma cells. 

To further explore the role of glutamine metabolism in metabolic reprogramming following CDK4/6 inhibition, we next monitored mitochondrial metabolism following treatment with palbociclib and the GLS1 inhibitor CB-839, a therapeutic drug currently in clinical trials [21,22]. The activity of CB-839 was first confirmed by a significant reduction in intracellular glutamate levels (Figure 3a). A significant decrease in maximal respiration and spare respiratory capacity was observed in both CB-839 and palbociclib combination-treated cell lines compared to palbociclib treatment alone (Figure 3b–d), indicating that the enhanced mitochondrial phenotype following CDK4/6 inhibition is partially dependent on glutamine metabolism. Importantly, CB-839 also increased the sensitivity of both cell lines to palbociclib in cell proliferation assays (Figure 3e). These results indicate that glutamine metabolism is functionally relevant with respect to drug sensitivity and suggest that glutamine metabolism plays a role in driving the enhanced mitochondrial phenotype following palbociclib treatment in melanoma cells. However, as mitochondrial metabolism was not restored to basal levels upon treatment with CB-839, this suggests that other metabolic pathways may also promote enhanced mitochondrial metabolism following palbociclib treatment. 

**Figure 4:**
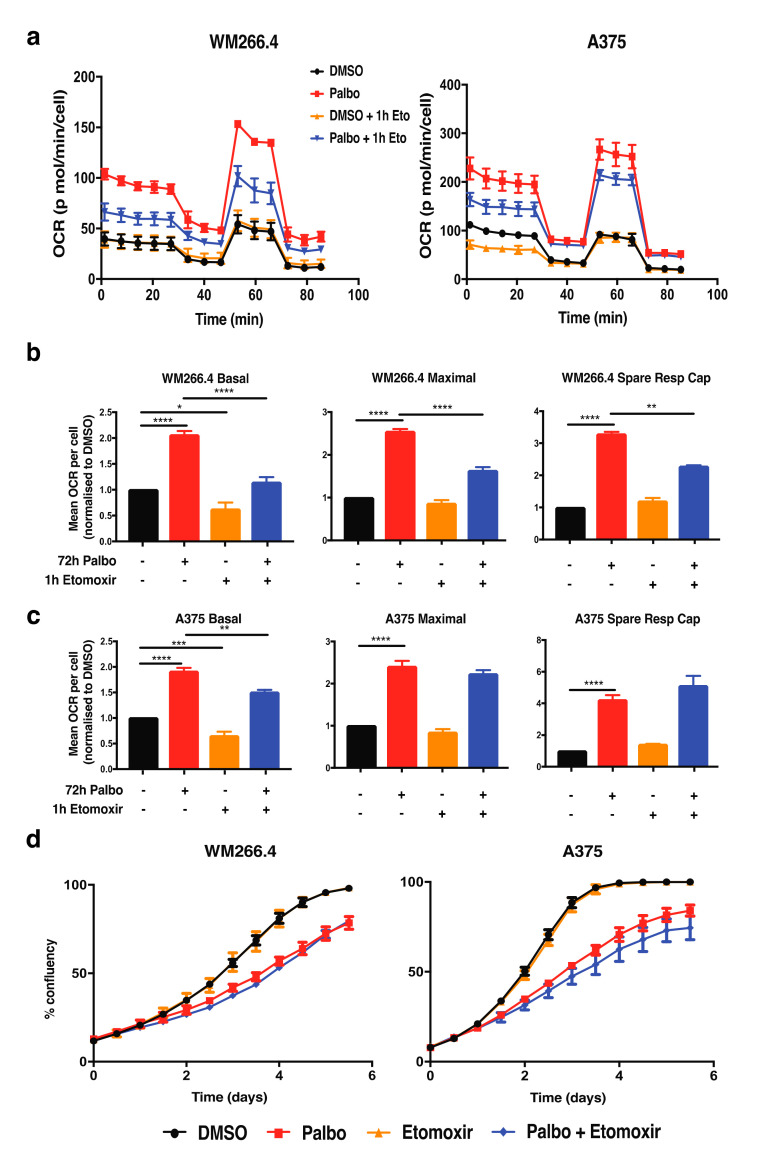
Increased mitochondrial metabolism following palbociclib treatment is partially dependent on fatty acid metabolism. (**a**) Oxygen consumption rate (OCR) was determined using Seahorse extracellular flux analysis in WM266.4 and A375 cells treated for 72 h with palbociclib (palbo; 1 μM) and etomoxir (Eto; 40 μM) as indicated. Data show a representative profile from 3 independent experiments. (**b**,**c**) Basal OCR, maximal OCR and spare respiratory capacity were determined from OCR profiles shown in (**a**) for WM266.4 (**b**) and A375 (**c**) cells. Data are normalised to cell number and expressed as fold change relative to DMSO controls. (**d**) WM266.4 and A375 cells were treated with palbociclib (1 μM) or etomoxir (40 μM) for 6 days, and percentage confluency was analysed over time using an IncuCyte to assess cell proliferation. Data are representative of 3 biological replicates. Error bars ± SEM, *n* = 3. Statistical significance was determined by One-way ANOVA: * *p* = 0.05–0.01, ** *p* = 0.01–0.001, *** *p* = 0.001–0.0001, **** *p* < 0.0001.

Interestingly, fatty acid oxidation has also been shown to play a role in CDK4 regulated metabolic reprogramming in other cancers [17]. Given that fatty acid oxidation also drives mitochondrial metabolism by feeding into the TCA cycle, the role of this pathway in metabolic reprogramming following CDK4/6 inhibition in melanoma cells was investigated. To determine whether the palbociclib-induced increase in mitochondrial metabolism was dependent on fatty acid oxidation, we utilised etomoxir, a specific inhibitor of CPT1, a mitochondrial transporter of long-chain fatty acids [23]. OCR values were observed following treatment of cells with etomoxir for 1 h and palbociclib for 72 h (Figure 4a). Notably, a significant decrease in basal OCR was observed in both cell lines following treatment with etomoxir alone for 1 h (Figure 4b,c), confirming activity of this inhibitor in melanoma cells. Etomoxir significantly decreased the palbociclib-induced increase in basal respiration, however a reduction in spare respiratory capacities was only observed in the WM266.4 cells (Figure 4b,c). Etomoxir alone had no effect on cell proliferation assays and did not alter the response to palbocilib (Figure 4d). Collectively, these observations indicate that although fatty acid oxidation plays a role in the metabolic response to palbociclib treatment in melanoma cells, this does not impact cell proliferation.

### 2.3. Adaptive Metabolic Responses to CDK4/6 Inhibition Are Partially Dependent on p53 in BRAF^V600^ Melanoma Cells 

Given the evidence of a role for both glutamine metabolism and fatty acid oxidation in metabolic reprogramming following CDK4/6 inhibition, we next focused on potential mechanisms underpinning this phenotype. Increased glutamine metabolism resulting from CDK4/6 inhibition in breast and colorectal cancer cells has been shown to be dependent on MYC-driven activation of mTOR [18]. Notably, the mTOR pathway can also activate fatty acid oxidation pathways [24]; thus, we explored the role of MYC and mTOR signalling in metabolic reprogramming following CDK4/6 inhibition in the context of melanoma cells. In contrast to what has been reported in breast and colorectal cancer cells [18], palbociclib induced a decrease in c-MYC expression, and no change in mTOR pathway activity, as assessed by p-S6 protein levels (Figure S4). Moreover, gene set enrichment analysis (GSEA) of a published RNA sequencing dataset [25] generated from A375 cells following treatment with palbociclib for 72 h revealed a decrease in MYC target genes (Figure S5a,b). Together, these data suggest that neither MYC nor the mTOR pathway is a key driver of enhanced mitochondrial metabolism following CDK4/6 inhibition in melanoma cells. Of note, GSEA also identified upregulation of the OXPHOS pathway consistent with the metabolic analyses reported here (Figure S5c). 

**Figure 5:**
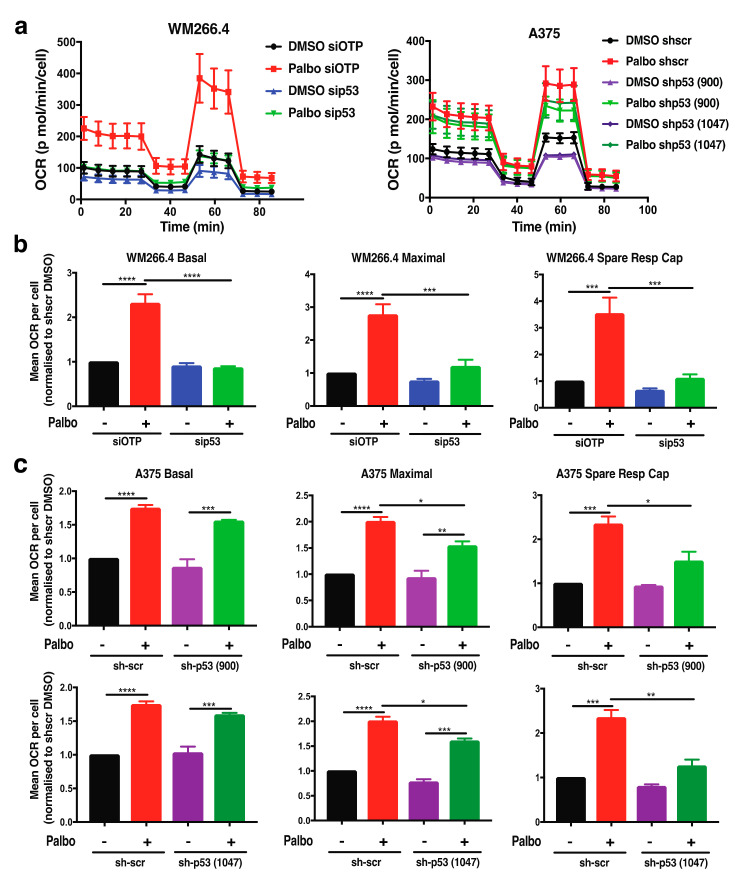
p53 regulates metabolic reprogramming following palbociclib treatment in melanoma cells. (**a**) Oxygen consumption rate (OCR) was determined using Seahorse extracellular flux analysis in WM266.4 and A375 cells treated for 72 h with palbociclib (palbo; 1 μM). Data show a representative profile from 3 independent experiments. (**b**,**c**) Basal OCR, maximal OCR and spare respiratory capacity were determined from OCR profiles shown in (**a**) for WM266.4 (**b**) and A375 (**c**) cells. Data are normalised to cell number and expressed as fold change relative to DMSO controls. Error bars ± SEM, *n* = 3. Statistical significance was determined by one-way ANOVA: * *p* = 0.05–0.01, ** *p* = 0.01–0.001, *** *p* = 0.001–0.0001, **** *p* < 0.0001.

We have previously demonstrated [25] that treatment with palbociclib activates the p53 pathway in melanoma cells, and our studies both confirm (Figure S5d) and extend this observation by demonstrating that p53 signalling is also activated in WM266.4 melanoma cells, as indicated by increased levels of phospho-p53 and p21 protein levels (Figure S6a). Notably, the majority of melanomas are wild-type for p53 [26], as are the A375 and WM266.4 cell lines. Further to roles in cell survival and death, p53 has also emerged as a critical mediator of metabolic pathways in response to nutrient stress [27,28]. Interestingly, this includes both glutamine metabolism and fatty acid oxidation [24]; therefore, we next explored the effect of p53 on palbociclib-induced metabolic reprogramming. In A375 cells, p53 knockdown was generated using shRNA as previously reported [25], while acute p53 knockdown in WM266.4 cells was attained using siRNA (Figure S6b). In WM266.4 cells, palbociclib induced an increase in both basal and maximal respiration, and importantly, spare respiratory capacity was reduced to control levels by p53 knockdown (Figure 5a,b). Although the effect of p53 knockdown was not as pronounced in A375 cells, the palbociclib induced increase in maximal respiration and spare respiratory capacity was significantly reduced by p53 knockdown using two independent shRNA constructs (Figure 5c). Together, these data demonstrate a role for p53 in metabolic reprogramming following CDK4/6 inhibition in the context of BRAF mutant melanoma cells.

Collectively, although CDK4/6 inhibition has no substantial effect on the metabolic phenotype following BRAF/MEK targeted therapy in melanoma, CDK4/6 inhibition alone significantly enhances mitochondrial metabolism. This increase in mitochondrial metabolism in melanoma cells following CDK4/6 inhibition is partially fuelled by both glutamine metabolism and fatty acid oxidation pathways and is mediated by a p53 dependent pathway. Collectively, our findings identify new p53 dependent metabolic vulnerabilities that may be targeted to improve response to CDK4/6 inhibitors. 

## 3. Discussion

With the success of CDK4/6 inhibitors in treating breast cancers [29,30], and likely other cancers in the near future [31,32], understanding their mechanism of action and interaction with other therapies is vital in their clinical development. In addition to regulating the cell cycle via phosphorylation and inhibition of the tumour suppressor RB, CDK4/6 are now known to phosphorylate numerous cellular proteins [33] and modulate many cellular functions including cell metabolism [34,35].

The current standard-of-care for BRAF mutant melanoma is a combination of BRAF and MEK inhibitors, and this combination with the addition of a CDK4/6 inhibitor induces sustained tumour regression in both BRAF and NRAS mutant melanoma preclinical models [10,11,36,37,38]. In this study, we demonstrate that although CDK4/6 inhibition does not potentiate the metabolic effects of BRAF and MEK inhibition in melanoma, there is a significant upregulation of mitochondrial activity following CDK4/6 inhibition. Importantly, we provide evidence of a role for both glutamine and fatty acid metabolism pathways in the enhanced mitochondrial phenotype following CDK4/6 inhibition, and we show this is mediated at least in part by the tumour suppressor p53.

BRAF/MEK inhibition reprograms metabolism in melanoma, involving a decrease in glycolysis [13] and a compensatory increase in oxidative phosphorylation that is mediated at least in part by a MITF-PGC1A pathway [14,39]. Our initial studies addressed the hypothesis that the triple BRAF/MEK/CDK4/6 combination in BRAF mutant melanoma cells would potentiate the metabolic effects of MAPK pathway inhibition and lead to enhanced suppression of glycolysis and/or increased OXPHOS and reactive oxygen species (ROS) production in melanoma cells. However, our investigation demonstrates that CDK4/6 inhibition does not significantly alter the effects of BRAF and MEK inhibitors on either glycolysis or OXPHOS in melanoma cells, nor does it significantly alter the expression of the MITF-PGC1A-mitochondrial metabolism pathway. These observations therefore suggest that the observed synergistic effects on melanoma cell proliferation and tumour growth of BRAF/MEK plus CDK4/6 inhibition occurs independently of the major metabolic pathways linked with response to BRAF and MEK targeted therapies.

The link between metabolism and cell-cycle regulators has garnered significant interest, and the role of CDK4/6 in metabolic reprogramming is an area of active investigation in multiple types of cancer [17,18,19]. Notably, the available evidence supports a multifaceted and context-dependent role for CDK4/6 in metabolism [34]. A role for CDK4 in glycolysis has been described in mouse embryonic fibroblasts (MEFs), whereby CDK4 knockout impairs anaerobic glycolysis [17], whilst the CDK4/6 inhibitor palbociclib reduces glycolysis in triple-negative breast cancer [40,41] and mesothelioma [42]. Glucose metabolism also appears to underpin synergistic activities of palbociclib in combination with PI3K/mTOR inhibitors in triple-negative breast cancer [41]. In contrast, both CDK4/6 knockdown and palbociclib enhanced glucose metabolism in HCT116 colorectal cancer cells [18], and elevated glycolysis was also observed in pancreatic cancer cells following CDK4/6 inhibition via palbociclib, abemaciclib and ribociclib [19]. Our analysis of BRAF mutant melanoma cells did not reveal any significant effect of CDK4/6 inhibition on glycolysis, nor did it alter the glycolytic response to BRAF/MEK inhibition, as indicated by no change in lactate production, extracellular acidification rate or expression of glycolysis pathway components. Thus, our data provide further evidence that the metabolic response to CDK4/6 inhibition is context-dependent. We also note that differences in palbociclib treatment regime (for example dose and treatment durations) may also underpin some of the observed differences in glucose metabolism reported in these studies.

A role for CDK4/6 in mitochondrial metabolism is also well supported, whereby increased oxidative mitochondrial metabolism is observed in pancreatic ductal adenocarcinoma cells treated with palbociclib [19], fuelled by increased glutamine consumption and metabolism. Notably, elevated OXPHOS was also induced by ribociclib and abemaciclib, indicating similar metabolic effects of different CDK4/6 inhibitors at least in the context of pancreatic cancer. CDK4/6 inhibition also increases mitochondrial metabolism in MEFs; however, in this case enhanced mitochondrial activity appeared to be fuelled by increased fatty acid oxidation [17]. Consistent with these studies, we observed increased mitochondrial activity and mass in melanoma cells following treatment with palbociclib, and our analysis supports a role for both glutamine and fatty acid oxidation pathways in this response. Treatment with palbociclib increased intracellular glutamate levels, and inhibition of glutamine metabolism using a glutaminase inhibitor CB-839 reduced the palbociclib-mediated increase in mitochondrial respiration, and importantly, this resulted in increased sensitivity to palbociclib. Our studies also identified a role for fatty acid metabolism in the metabolic response to palbociclib, whereby inhibition of fatty acid metabolism using the CPT1 inhibitor etomoxir also reduced the palbociclib-induced mitochondrial phenotype; however, this did not significantly impact cell proliferation. Together, these observations suggest that CDK4/6 inhibition reprograms mitochondrial metabolism in melanoma cells via both glutamine and fatty acid-oxidation-dependent pathways; however, only reprogrammed glutamine metabolism appears significant with regard to CDK4/6 inhibitor sensitivity and the proliferative response.

Mechanistically, increased glutamine metabolism resulting from CDK4/6 inhibition in breast and colorectal cancer cells has been shown to be dependent on MYC-driven activation of mTOR [18]. However, analysis of A375 melanoma cells after treatment with palbociclib revealed significant downregulation of MYC target genes, and no change in mTOR signalling or c-MYC protein, suggesting that the mechanism in melanoma cells is distinct from colorectal cancer cells. We have recently shown that CDK4/6 inhibition activates the p53 tumour suppressor in melanoma cells by reducing expression of the p53-inhibitor MDM4 [25], and here we extend this observation by demonstrating a role for p53 in the adaptive metabolic response to CDK4/6 inhibition. Although p53 functions as a critical tumour suppressor that contributes to cell death and cell cycle arrest in response to various stresses, intriguingly, in the context of metabolic stress, p53 triggers a primarily adaptive rather than pro-apoptotic response [27,43]. Indeed, our observations are consistent with emerging evidence describing a role for p53 in mediating cancer cell adaptation and survival responses to different types of metabolic stress in a cell type and stimuli-specific manner [28,43,44,45,46]. Specifically, p53 promotes adaptive survival responses to glutamine deprivation in both wild-type and RAS-transformed MEFs, triple-negative breast cancer, pancreatic cancer and human lymphoma cell lines. Importantly, our data now suggest a role for p53 in metabolic reprogramming in response to perturbations in cell cycle regulators in the context of melanoma cells that are wild type for p53.

In summary, our metabolic analyses have revealed that inhibition of CDK4/6 upregulates mitochondrial function in BRAF mutant melanoma cells. Elevated mitochondrial metabolism is fuelled by both glutamine and fatty acid metabolism and occurs via a p53-dependent mechanism. Further investigations are required to identify the key transcriptional targets of p53 that drive this response, and also determine whether the CDK4/6 driven metabolic phenotype is conserved across additional mutational sub-types of melanoma. Although there are similarities between the effects of the distinct CDK4/6 inhibitors with regard to glycolysis and OXPHOS in some cancer models [19], there may also be drug-specific effects on metabolism [34], which also warrants further investigation. Such studies would thus determine the potential of combining CDK4/6 inhibitors with inhibitors of adaptive metabolic pathways, such as glutaminase inhibitors, as a future therapeutic option for melanoma patients that do not respond to currently available treatments.

## 4. Materials and Methods

### 4.1. Cell Lines

WM266.4 and A375 BRAF mutant melanoma cells were purchased from the American Type Culture Collection (ATCC). All melanoma cell lines were cultured using RPMI 1640 medium, with 10% (*v*/*v*) Fetal Bovine Serum (FBS) and 2 mM Glutamax (Life Technologies 35050-061, California, CA, USA), and were maintained in a 37 °C humidified, 5% CO_2_ incubator. The identity of all cell lines was confirmed using STR profiling. Stable p53 knockdown A375 cell lines were generated previously [26].

### 4.2. Pharmacologic Inhibitors

Vemurafenib (PLX4032, S1267), Palbociclib (PD-0332991) and Cobimetinib were purchased from Selleckchem (Houston, TX, USA. Antimycin A (A8675), Etomoxir (E1905), Carbonylcyanide p-trifluoromethoxyphenylhydrazone (FCCP, C2920), Oligomycin (75351) and Rotenone (R8875) were purchased from Sigma-Aldrich (Castle Hill, New South Wales, Australia). CB-839 was a generous gift from Calithera Biosciences (South San Francisco, CA, USA) (Table 1).

### 4.3. Western Immunoblotting

Protein was extracted from cells using western solubilization buffer (WSB; 0.5 mM EDTA, 20 mM HEPES, 2% SDS) and boiled for 10 min at 95 °C unless otherwise specified. Protein samples were subjected to SDS-PAGE analysis followed by western immunoblotting using the following antibodies: ERK (p44/42-MAPK), phospho-ERK (p44/42-MAPK; Thr202/Tyr204), HK2, p21(12D1), phospho-p53 (Ser15), phospho-RB (Ser780), phospho-S6 (Ser240/244) from Cell Signalling Technologies; GLS1, HIF1α, c-MYC, OXPHOS cocktail from Abcam; MITF (Merck Millipore, Dermadst, Germany), p53 (DO1) (Santa Cruz Biotechnology, Dallas, Texas, USA), PDHA1 (AbNova, Taipei, Taiwan), phospho-PDHE1 (Ser293) (Novus Biologicals, Colorado, USA), RB (BD Biosciences, California, USA). Primary antibodies were used at 1:1000, and secondary antibodies (Sigma-Aldrich, Missouri, United States) conjugated with horseradish peroxidase (HRP) were used at 1:2000 in 1× TBS-T containing 5% (*w*/*v*) skim milk powder (Diploma Skim Milk Powder, Bonlac, Melbourne, Australia). For analysis of OXPHOS proteins using the OXPHOS antibody cocktail, samples were boiled for 10 min at 50 °C as per manufacturer’s directions, and SDS-PAGE analysis was performed using an 8–16% Mini PROTEAN TGX Gel (Biorad, California, CA, USA). Densitometry analysis of proteins was performed using ImageJ, and bands of interest were normalised to the intensity of the corresponding loading control.

### 4.4. qRT-PCR

RNA was harvested and isolated following the RNeasy MiniKit standard protocol (QIAGEN 74104). The concentration of RNA (ng/μL) was determined by a NanoDrop ND-1000 analyser (Analytical Technologies, Pennsylvania, USA). qRT-PCR was performed by preparing a master mix containing 30% cDNA sample, 50% Fast SYBR Green Master Mix (Applied Biosystems 4385612), and 0.1 μM forward and reverse oligo-primers, which were plated in triplicate. qRT-PCR reactions were run using the StepOnePlus Real-Time PCR Platform (Applied Biosystems, Foster City, CA, USA), initially at 95 ℃ for 20 s, then 40 cycles of 95 ℃ for 3 s and 60 ℃ for 30 s. Data were processed using the comparative CT method, relative to the housekeeping gene NONO (Table 2). Changes in mRNA expression were expressed as Log2 fold change relative to DMSO controls and analysed using a Students *t*-test or one-way ANOVA (*p* < 0.05).

### 4.5. Lactate Assay

Cells were seeded into black-walled 96-well plates in phenol-free RPMI 1640 media (Gibco, 11835-030, Thermo Fisher Scientific, Waltham, MA, USA), including media-only wells to be used for background subtraction. Cells were treated with inhibitors 24 h post-seeding. At completion of the drug treatment, plates were centrifuged for 5 min at 500× *g,* and growth media were collected. Media were diluted 1:3 with PBS and snap frozen at −80 °C. Lactate levels were determined using an L-lactate assay kit (Eton Biosciences, California, CA, USA), as per manufacturer’s instructions. Absorbance was determined using a Cytation 3 Imaging Multi-Mode plate reader (Biotek, Vermont, VT, USA). After media collection, cells were fixed and stained with DAPI DNA dye and cells were imaged using a Cellomics Arrayscan automated microscope. Image analysis and cell number calculation was performed using the Cellomics “Cell cycle” bioapplication (10× magnification; 16× fields). Background absorbance from media-only wells was subtracted, and data were normalised to cell number to generate the parameter lactate production per cell.

### 4.6. Extracellular Flux Analysis

Extracellular flux analyses were performed on a Seahorse XF^e^96 Analyzer (Agilent, Santa Clara, California, CA, USA). For all assays, Flux Packs that contained the cell culture microplates, sensor cartridges and XF calibrant were used (Agilent 102416-100). Assay medium was prepared using Seahorse XF Base Medium DMEM (containing 5.5 mM glucose, 2 mM glutamine and 1 mM sodium pyruvate, adjusted to pH 7.4 and kept at 37 °C). Prior to cell seeding, the cell culture microplate was coated with Corning Cell-Tak (438512) under sterile conditions, as per manufacturer’s directions. After the desired duration of gene knockdown and drug treatment, cell culture medium was removed and replaced with Seahorse XF medium, and cells were equilibrated in a non-CO_2_ incubator for 1 h prior to the assay. The XF Cell Mito Stress Test protocol was performed as per manufacturer’s directions, using oligomycin (1 μM), FCCP (1 μM) and rotenone/antimycin A (0.5 μM). The assay was run with repeated cycles of 3 min mix and 3 min measurements for 5 cycles to establish the basal metabolic phenotype and 3 cycles following injection of each drug with simultaneous measurement of oxygen consumption rate (OCR) and extracellular acidification rate (ECAR). At completion of the assay, cells were injected with Hoescht live-cell nuclear stain and imaged using a Cellomics Arrayscan automated microscope (10× magnification; 4× fields). Image analysis and cell number calculation were performed using the Cellomics “Cell cycle” bioapplication as described above. OCR and ECAR values were subsequently normalised to cell number, and data were analysed using the Mito Stress Test Report Generator (Agilent).

### 4.7. Glutamate Detection Assay

Cells were seeded in a 96-well plate (Falcon) in 100 μL medium per well, and 24 h post-seeding, 50 μL of drug-containing medium was added to each well, in triplicate. Additional wells were seeded and treated in parallel to allow calculation of cell number. After 72 h treatment, cells were harvested and processed using the Promega Glutamine-Glutamate-Glo^TM^ Assay protocol, as per manufacturer’s directions (Promega, Wisconsin, WI, USA). Luminescence was determined using a Cytation 3 Imaging Multi-Mode plate reader (Biotek). To calculate cell number, cells were fixed and stained with DAPI DNA dye and imaged using a Cellomics Arrayscan automated microscope. Image analysis and cell number calculation were performed using the Cellomics “Cell cycle” bioapplication (10× magnification; 16× fields).

### 4.8. Mitotracker Assay

Cells were seeded in a 6-well plate and drug-treated as indicated 24 h post-seeding. After 72 h treatment, growth media was replaced with Mitotracker RED dye (Invitrogen, California, CA, USA) prepared in serum-free media (400 nM) and incubated for 30 min at 37 °C. Cells were trypsinized and transferred to FACs tubes and washed with PBS. Fix yellow (Invitrogen) was added to cells and incubated for a further 30 min in order to assess cell viability. Cells were then washed and fixed with 4% paraformaldehyde prior to analysis on a BD FACSymphony analyser. Mitochondrial mass and activity were determined as the median fluorescence intensity.

### 4.9. siRNA Mediated Gene Knockdown

Cells were forward-transfected with 40 nM siGENOME SMARTpool siRNAs (Dharmacon) using 0.08 μL of Lipofectamine^TM^ RNAiMAX (Invitrogen) per 100 μL of transfection media per well, as per manufacturer’s directions. Briefly, RNAiMAX transfection lipid was diluted in OPTIMEM and equilibrated for 5 min, prior to complexing with siRNA for 20 min at room temperature. A non-targeting siOTP-NT siRNA was used as a control. Media were changed 24 h after transfection and plates were incubated at 37 °C for indicated times and/or drug-treated as described.

### 4.10. Proliferation and Viability Assays

Cells were plated in 96-well plates at low density and treated with medium containing inhibitors 24 h post-seeding. Phase-contrast images were acquired and analysed every 12 h, up to 6 days, using the IncuCyte (Essen Bioscience, Michigan, MI, USA) continuous live-cell imaging and analysis system. To assess viability, media were supplemented with propidium iodide (PI; 1 μg/mL) and PI stained cells were counted using automated image analysis and corrected for cell confluency.

### 4.11. Drug Dose–Response Assays

Dose–response assays were conducted in 96-well plates following either 72 h (vemurafenib and cobimetinib) or 6 day (palbociclib) drug treatments. Cells were fixed and permeabilized with methanol (MetOH), stained with DAPI nuclear dye and imaged using a Cellomics Arrayscan automated microscope (10× magnification; 16× fields). Image analysis and cell number calculation was performed using the Cellomics “Cell cycle” bioapplication. Log[inhibitor] vs. response curves were generated by non-linear regression/curve fitting, and GI50 concentrations (the concentration of drug required to reduce growth by 50%) were obtained as a measure of drug sensitivity.

### 4.12. Gene Set Enrichment Analysis (GSEA)

GSEA was performed on our previously published RNA sequencing dataset generated from A375 cells treated with palbociclib for 72 h [25]. Genes were ranked based on Log2FC normalized for the adjusted *p*-value (Log2FCx1/adjp-val) and run against the Hallmark (V6.2) gene sets using the preranked GSEA tool within the GSEA 4.0.3 software (Broad Institute, Massachusetts, MA, USA). Gene sets with FDR < 0.05 were considered significant.

## 5. Conclusions

Our analyses have revealed that inhibition of CDK4/6 upregulates glutamine and fatty acid-oxidation-dependent mitochondrial metabolism in BRAF mutant melanoma cells, but they do not alter glycolysis nor the metabolic response to MAPK inhibitors. Elevated mitochondrial metabolism in CDK4/6 inhibited cells occurs via a p53-dependent mechanism in melanoma cells that are wild-type for p53, extending the context-dependent role of CDK4/6 in cellular metabolism. These observations shed light on how CDK4/6 inhibitors impinge on the regulation of metabolism and how they interact with other therapies in the setting of melanoma.

## Figures and Tables

**Table 1:** Drug concentrations used for proliferation, viability and metabolism assays.

Drug Treatment	Proliferation + Viability Assays	Metabolism Assays
PLX (Plx4032, Vemurafenib)	300 nM	1 μM
Palbo (PD-0332991, Palbociclib)	1 μ μM	1 μM
Cobimetinib	1 nM	10 nM
PLX/Cobimetinib	300 nM/1 nM	1 μM/10 nM
PLX/Palbo/Cobimetinib	300 nM/1 nM/1 μM	1 μM, 1 μM, 10 nM (respectively)
Oligomycin	1 μM	1 μM
CB-839	200 nM	200 nM
Etomoxir	40 μM	40 μM

**Table 2:** qRT-PCR Primer Sequences.

Gene	Direction	Sequence
c-Myc	Forward	GGACGACGAGACCTTCATCAA
	Reverse	CCAGCTTCTCTGAGACGAGCTT
GLUT1	Forward	TCTCTGTGGGCCTTTTCGTT
	Reverse	CAGTTTCGAGAAGCCCATGAG
GLUT3	Forward	GGTGGAAGTACGTTATTGTTGACTTATT
	Reverse	GTTTGGCTAAAGGGTCTGAGATGT
HK2	Forward	AAGGCAATAGGGCCTTAAAGTAGAG
	Reverse	TTCGAGGCTGCAGTGAGCTA
HIF1α	Forward	TTTACCATGCCCCAGATTCAG
	Reverse	GGTGAACTTTGTCTAGTGCTTCCA
MITF	Forward	CCGTCTCTCACTGGATTGGT
	Reverse	TACTTGGTGGGGTTTTCGAG
NONO	Forward	CATCAAGGAGGCTCGTGAGAAG
	Reverse	TGGTTGTGCAGCTCTTCCATCC
PGC1a	Forward	CTGCTAGCAAGTTTGCCTCA
	Reverse	AGTGGTGCAGTGACCAATCA
TFAM	Forward	TACCGAGGTGGTTTTCATCTG
	Reverse	AACGCTGGGCAATTCTTCTA

## Data Availability

All source data relating to this manuscript are available upon request.

## Supplementary Materials

The following are available online at https://www.mdpi.com/2072-6694/13/3/524/s1, Figure S1: Assessment of palbociclib (Palbo), vemurafenib (Vem) and cobimetinib (Cobi) efficacy; Figure S2: Palbociclib (Palbo) treatment has no substantial effect on the metabolic phenotype of vemurafenib (Vem) and Cobimetinib (Cobi); Figure S3: Palbociclib (Palbo) treatment results in increased mitochondrial respiration; Figure S4: Increased mitochondrial metabolism following palbociclib treatment occurs independently of changes in glutaminase levels, or Myc and mTOR pathway activity in melanoma cells; Figure S5: Palbociclib (palbo) treatment upregulates the oxidative phosphorylation and p53 pathway in A375 cells; Figure S6: Palbociclib (palbo) treatment upregulates the p53 pathway in A375 and WM266.4 melanoma cells.
