# Supplementary material for: CDK4/6 Inhibition Reprograms Mitochondrial Metabolism in BRAFV600 Melanoma via a p53 Dependent Pathway
Source: Cancers (Basel). 2021 Jan 29;13(3):524. doi: 10.3390/cancers13030524 (PMC7866416; doi:10.3390/cancers13030524)

Supplementary Figure Legends

Figure S1. Assessment of palbociclib (Palbo), vemurafenib (Vem) and cobimetinib (Cobi) efficacy.
(a) WM266.4 and A375 cells were treated as indicated for 72h and protein lysates were assessed for the indicated proteins using western blot analysis. Data is representative of 3 biological replicates. **(b)** Dose response assays for the indicated inhibitors were performed to establish the sensitivity of WM266.4 and A375 cells to each drug. Data is representative of 3 biological replicates. **(c)** Table summarising the mean GI50 for each inhibitor in the WM266.4 and A375 cells (N=3). **(d)** WM266.4 and A375 cells were treated for 6 days as indicated, and percent confluency analysed over time using an IncuCyte to assess cell proliferation. Data is representative of 3 biological replicates. **(e)** WM266.4 and A375 cells were treated for 72hr as indicated and assessed for cell death using propidium iodide (PI) staining. PI cell counts were normalised to cell confluency. Error bars $\pm$ SEM, N=3. Statistical significance was determined by One-way ANOVA: * P = 0.05-0.01, ** P = 0.01-0.001, *** P = 0.001-0.0001, **** P <0.0001.
Figure S2. Palbociclib (Palbo) treatment has no substantial effect on the metabolic phenotype of vemurafenib (Vem) and Cobimetinib (Cobi).
(a) Quantitative realtime PCR (Q-RT-PCR) gene expression analysis of the glycolysis pathway and regulators of mitochondrial biogenesis in WM266.4 and A375 cells treated as indicated for 72h. Data is expressed as Log2 fold change relative to DMSO controls (n=3-4 biological replicates). **(b)** WM266.4 and A375 cells were treated as indicated for 72h and protein lysates were assessed for the indicated proteins using western blot analysis. Data is representative of 3 biological replicates.
Figure S3. Palbociclib (Palbo) treatment results in increased mitochondrial respiration.
(a-b) Representative oxygen consumption rate (OCR) profiles determined using Seahorse extracellular flux analysis in WM266.4 **(a)** and A375 **(b)** cells treated as indicated for 72h. Cells were treated with mitochondrial inhibitors (oligomycin, FCCP and antimycin A/rotenone) to determine basal respiration, maximal respiration, and spare respiratory capacity (see methods for details). Data is normalised to cell number.
Figure S4. Increased mitochondrial metabolism following palbociclib treatment occurs independently of changes in glutaminase levels, or Myc and mTOR pathway activity in melanoma cells.
(a) WM266.4 and A375 cells were treated as indicated for 48h, 72h, and 96h, and protein lysates were assessed for glutaminase (GLS1) protein using western blot analysis. GLS1 levels were quantified across 3 independent experiments (bottom panel). **(b)** WM266.4 and A375 cells were treated as indicated for 48h, 72h, and 96h, and lysates were assessed for the indicated proteins using western blot analysis. Data is representative of n=3 biological replicates.
Figure S5. Palbociclib (palbo) treatment upregulates the oxidative phosphorylation and p53 pathway in A375 cells.
(a) Gene set enrichment analysis (GSEA) was performed on a published RNA sequencing dataset of A375 cells treated with palbo for 72hr[25]. The normalised enrichment score (NES) of significant hallmark gene sets are shown (FDR < 0.05). **(b-d)** GSEA profiles for the hallmark gene sets MYC targets **(b)**, oxidative phosphorylation **(c)**, and p53 pathway **(d)**.

Figure S6. Palbociclib (palbo) treatment upregulates the p53 pathway in A375 and WM266.4 melanoma cells.

(**a**) WM266.4 and A375 cells were treated as indicated for 48h and 72h, and protein lysates were assessed for p53 pathway activity using western blot analysis. **(b)** Knockdown of p53 protein in WM266.4 cells (left panel) and A375 cells stably expressing p53 shRNA (900 & 1047) (right panel) was determined using western blot analysis[25].

Figure S1

A.

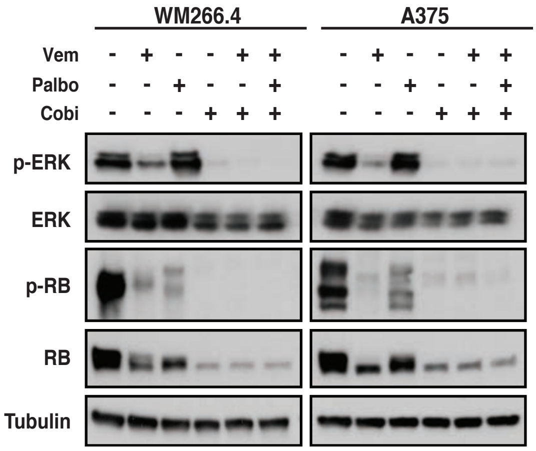

B.

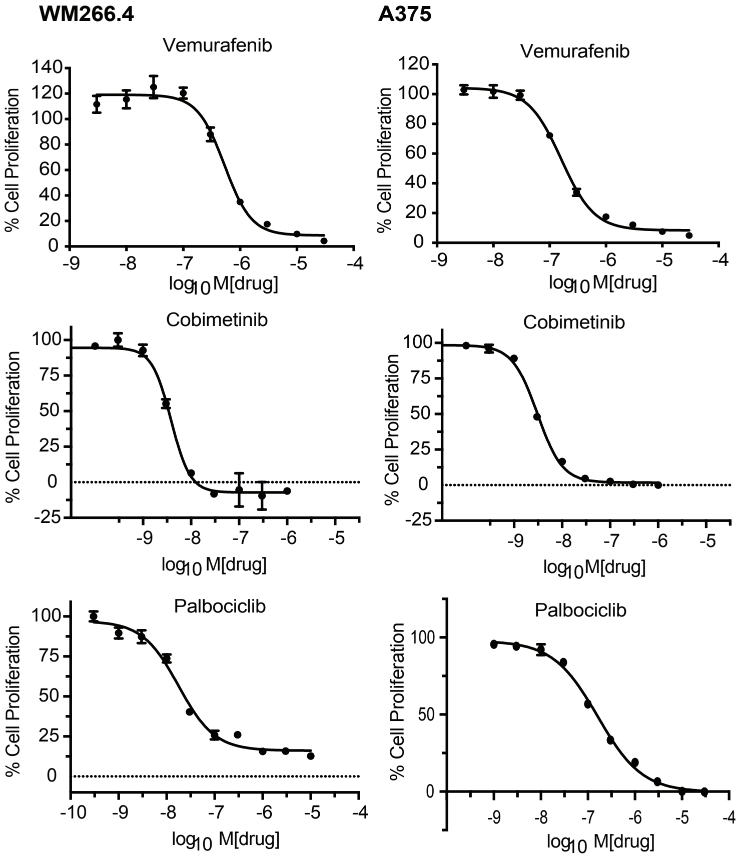

C.

Drug	A375 (GI50)	WM266.4 (GI50)
Vemurafenib	16nM	53.1nM
Cobimetinib	3nM	3.4nM
Palbociclib	107nM	18.2nM

D.

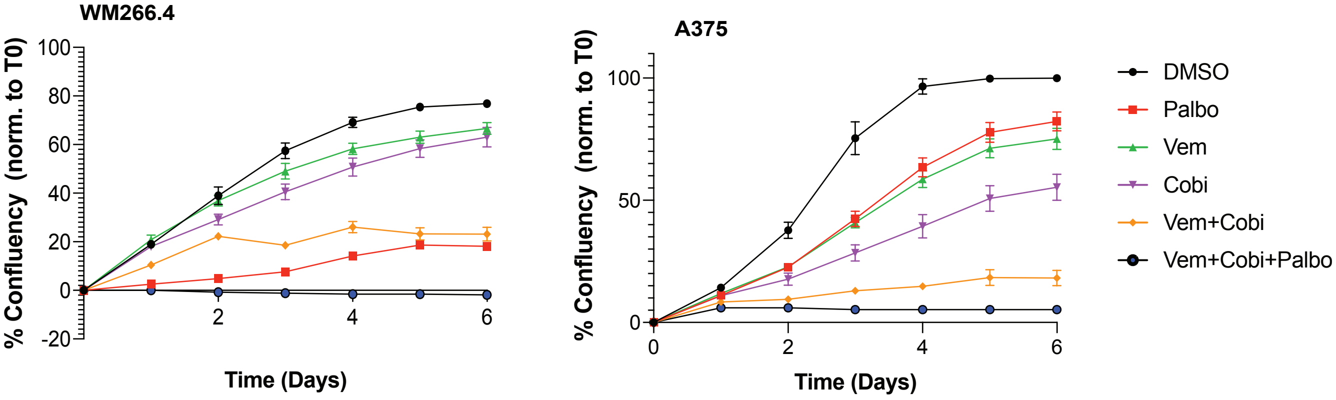

E.

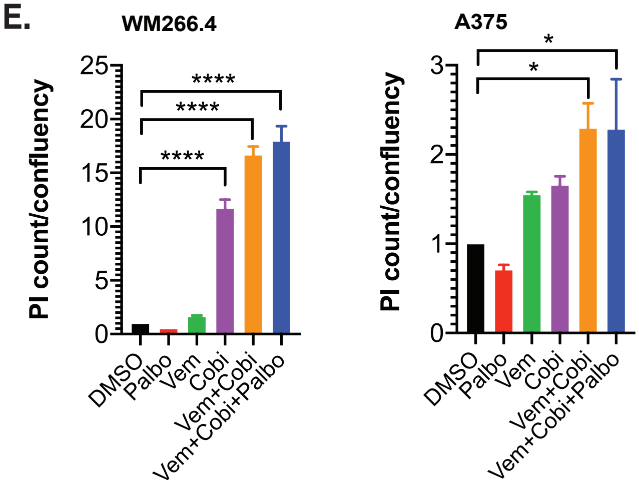

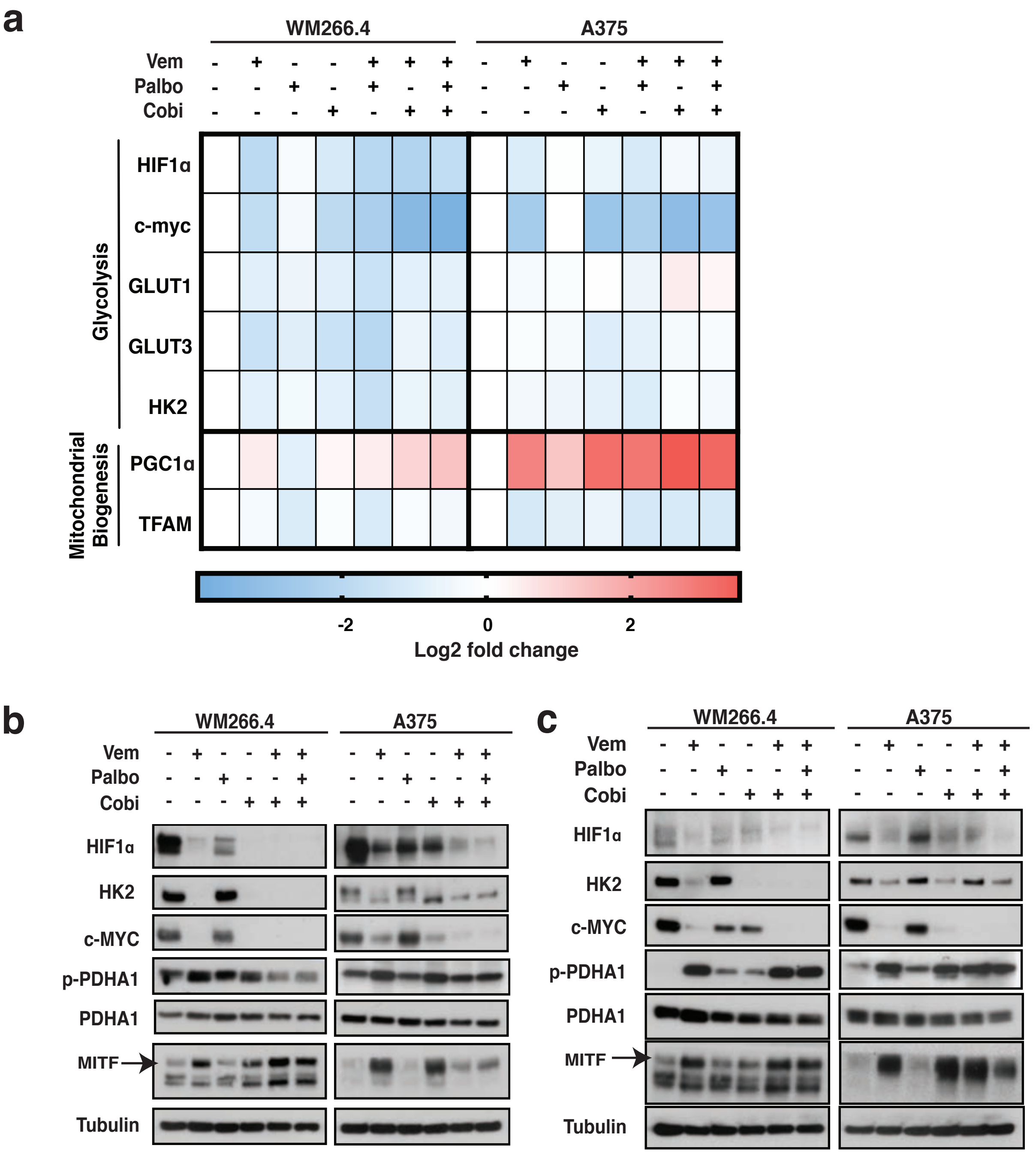

a
WM266.4
A375
Vem
Palbo
Cobi
HIF1α
c-myc
GLUT1
GLUT3
HK2
PGC1α
TFAM
Glycolysis
Mitochondrial Biogenesis
-2
0
2
Log2 fold change
b
WM266.4
A375
Vem
Palbo
Cobi
HIF1α
HK2
c-MYC
p-PDHA1
PDHA1
MITF
Tubulin
c
WM266.4
A375
Vem
Palbo
Cobi
HIF1α
HK2
c-MYC
p-PDHA1
PDHA1
MITF
Tubulin

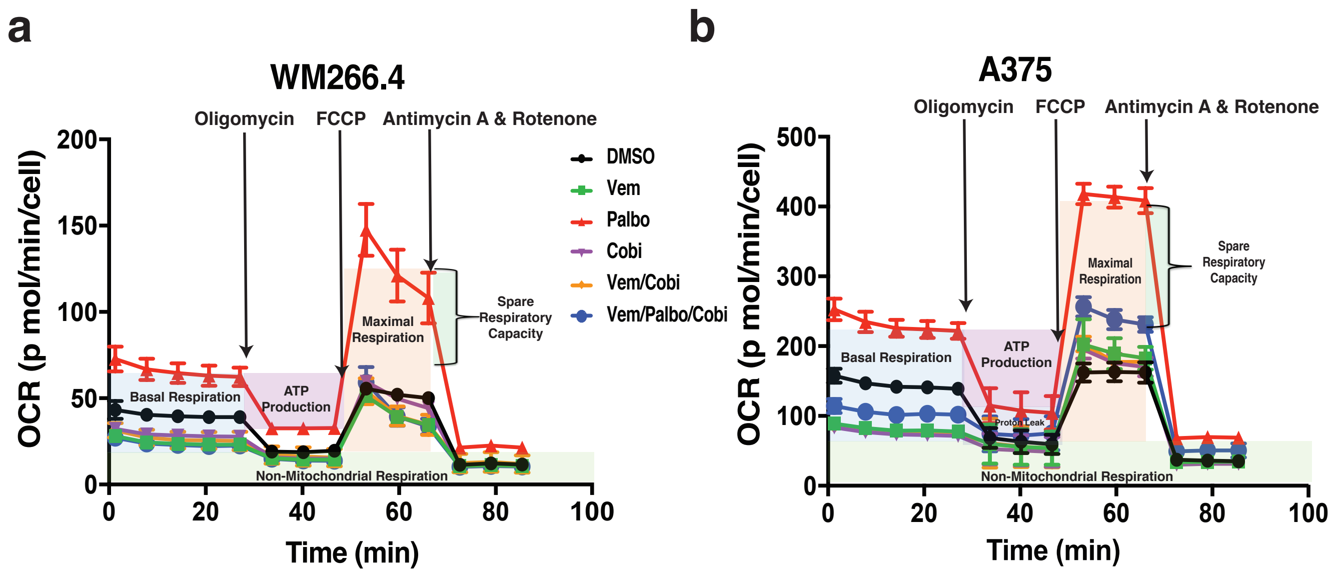
a
WM266.4
Oligomycin
FCCP
Antimycin A & Rotenone
OCR (p mol/min/cell)
Time (min)
Basal Respiration
ATP Production
Maximal Respiration
Spare Respiratory Capacity
Non-Mitochondrial Respiration
DMSO
Vem
Palbo
Cobi
Vem/Cobi
Vem/Palbo/Cobi
b
A375
Oligomycin
FCCP
Antimycin A & Rotenone
OCR (p mol/min/cell)
Time (min)
Basal Respiration
ATP Production
Proton Leak
Maximal Respiration
Spare Respiratory Capacity
Non-Mitochondrial Respiration

a

b

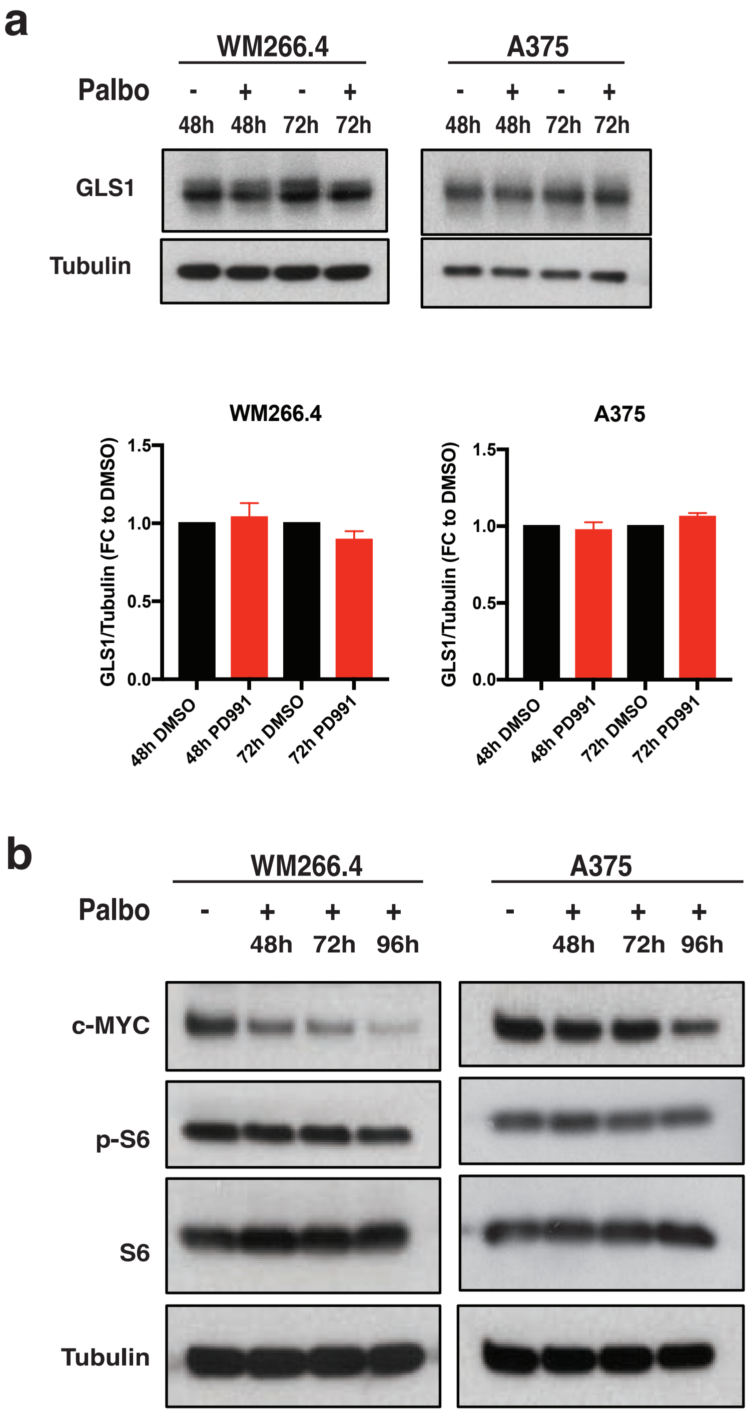
WM266.4
A375
Palbo
- + - +
48h 48h 72h 72h
GLS1
Tubulin
WM266.4
A375
GLS1/Tubulin (FC to DMSO)
1.5
1.0
0.5
0.0
48h DMSO
48h PD991
72h DMSO
72h PD991
WM266.4
A375
Palbo
- + + +
48h 72h 96h
c-MYC
p-S6
S6
Tubulin

Supplementary Figure S5

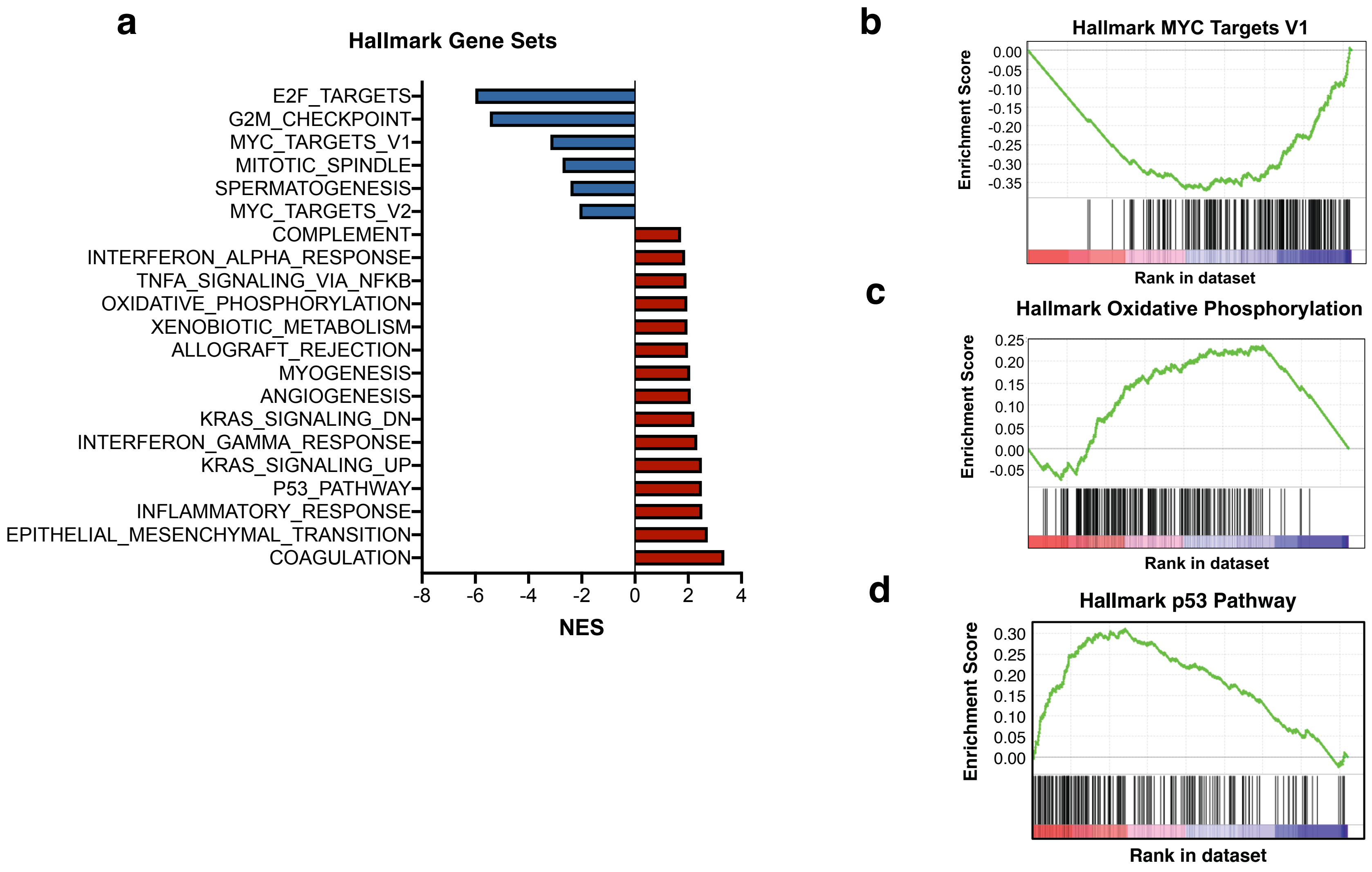

Supplementary Figure S6

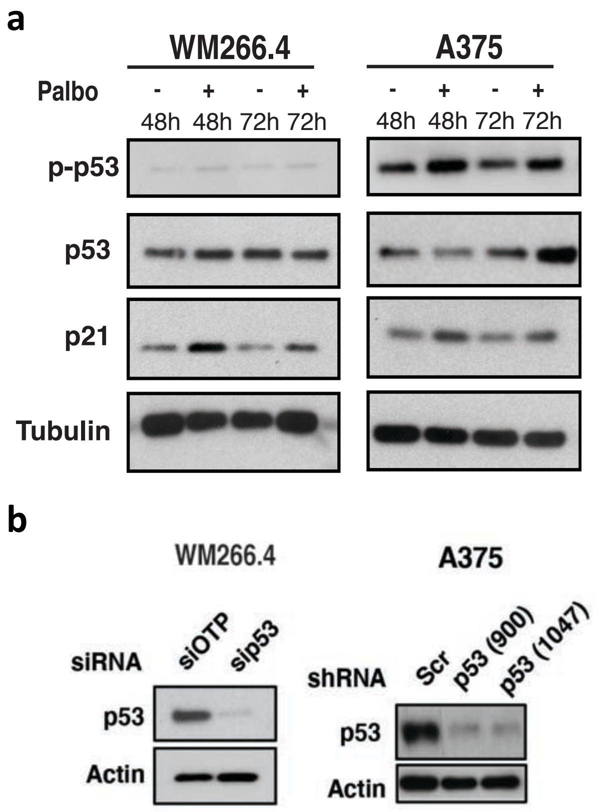

Supplement: Supplementary file 1 [file cancers-13-00524-s001.pdf]
